# Biomechanical risk factors for rotator cuff syndrome in high-risk occupations: A prospective study protocol

**DOI:** 10.1371/journal.pone.0326229

**Published:** 2025-06-20

**Authors:** Angelica E. Lang, Kenzie B. Friesen, Josh Lawson, Prosanta Mondal, Niels Koehncke, Soo Y. Kim, Philip Chilibeck

**Affiliations:** 1 Canadian Centre for Rural and Agricultural Health, University of Saskatchewan, Saskatoon, Canada; 2 Department of Medicine, College of Medicine, University of Saskatchewan, Saskatoon, Canada,; 3 Department of Community Health and Epidemiology, College of Medicine, University of Saskatchewan, Saskatoon, Canada; 4 School of Rehabilitation Sciences, College of Medicine, University of Saskatchewan, Saskatoon, Canada; 5 College of Kinesiology, University of Saskatchewan, Saskatoon, Canada; Manipal College of Health Professions, INDIA

## Abstract

**Background:**

Rotator cuff syndrome (RCS) is the most common upper limb musculoskeletal disorder worldwide. RCS negatively impacts quality of life and comes with high costs to the individual and society through time loss of work or healthcare usage. Identifying modifiable risk factors for RCS is a critical avenue for exploration to improve prevention and treatment of RCS.

**Objective:**

The overarching goal of this research is to explore the connection between shoulder kinematics and RCS in high-risk occupations and determine if pre-injury shoulder kinematics during a standardized overhead reaching motion are a risk factor for symptomatic RCS.

**Methods:**

A prospective cohort design will be used to assess 292 individuals who work in high-risk occupations, such as construction, farming, and healthcare. Workers without any shoulder pain or disorders will be asked to attend an in-laboratory baseline testing session. First participants will complete questionnaires about their baseline symptoms, personal characteristics, and work exposures. They will then perform a standardized functional reaching task while their shoulder movement is tracked with optical motion capture. Participants will be surveyed every 3 months for two years; individuals with any indications of shoulder symptoms that develop during the study period will be further assessed with clinical impingement tests. Logistic regression and survival analyses will be performed to determine if scapular kinematics pre-injury, combined with several individual and work-related factors, are a risk factor for development of RCS.

**Proposed Results:**

These findings will provide empirical evidence to clarify the contribution of biomechanics to injury development. Specifically, it is expected that scapular kinematics at the baseline assessment will be a risk factor for the development of RCS.

**Conclusions:**

This research represents a crucial step for understanding shoulder musculoskeletal health. This information is foundational for development of innovative, evidence-based treatment and prevention strategies.

## Introduction

Musculoskeletal disorders are one of the leading causes of morbidity in the world [1]. Injuries and disorders of the upper extremity affect an estimated 16% of the global population and are the second most common musculoskeletal problem worldwide [2]. In Canada, upper limb musculoskeletal injuries make up approximately 20% of all Worker’s Compensation Board claims, substantially burdening the workforce [3]. Repeated cumulative trauma disorders, such as carpal tunnel, elbow pain, and shoulder pain, are responsible for the majority of serious upper limb injuries [4].

One of the most common cumulative trauma disorders of the upper limb includes injury or damage to the rotator cuff tendons [5]. This type of musculoskeletal disorder is often termed rotator cuff syndrome (RCS), which for this manuscript will encompass all symptomatic injury or degenerative conditions affecting the rotator cuff-related anatomical structures, such as subacromial impingement syndrome, bursitis, rotator cuff tendonitis, or partial or full-thickness rotator cuff tears [6]. RCS accounts for up to 65–70% of all shoulder disorders [7]. Direct and indirect costs of RCS are estimated to be up to $32,000 per case [8] suggesting that the economic burden of these upper limb disorders may be over one billion dollars per year in Canada. Identifying the risk factors for RCS and intervening early, therefore, is imperative for overall musculoskeletal health and reducing socio-economic consequences. RCS also has a significant negative impact on quality of life due to pain, reduced abilities or capacity, and even sleep interruptions [9–11] Understanding how individual movement strategies, a potentially modifiable factor, influence RCS development will enhance the ability to prevent and address RCS and improve quality of life.

While RCS is among the most common upper limb repetitive strain injuries, and certain work exposures increase the likelihood of RCS, little data exist on the specific incidence of new cases of RCS in high-risk occupations. Additionally, more information on modifiable risk factors that influence injury occurrence is sorely needed [12]. For instance, the prevalence of RCS is higher in workers with high ergonomic or overhead exposures (such as construction workers), but it is not well explained by frequency of movement, lack of pauses, or force requirements [13]. While it is well established that repetitive arm elevation increases the likelihood of developing RCS [14], it is not clear why some workers with these exposures are injured and others are not. Instead, it may be the exposures in these high-risk occupations combined with individual movement patterns that are responsible for injury.

There are key biomechanical components to cumulative trauma musculoskeletal injuries that are crucial to improve prevention and treatment of these disorders. For RCS in particular, a strong relationship between select scapular movement alterations is present in individuals already diagnosed with RCS [15,16]. However, these relationships have notably only been studied in an already injured population. To our knowledge, there have been no known prospective assessments of shoulder biomechanics and their relationship to RCS during functional tasks, which could more conclusively identify which movement strategies, if any, are related to the cause of the injury. This will provide unprecedented information regarding both the burden of RCS in the working population and possible risk factors for injury.

The overarching goal of this research is to improve understanding of the biomechanical contributions of RCS development. Findings will guide future research towards novel prevention and rehabilitation strategies to ultimately reduce the high burden of shoulder disorders.

The primary research question is:

1. Are pre-injury shoulder kinematics during a standardized overhead reaching motion a risk factor for symptomatic RCS?

It is expected that scapular kinematics at baseline measurement will be a risk factor for the development of RCS in the study period. Specifically, scapular kinematics that are associated with the presence of RCS and considered harmful, such as reduced upward rotation and increased internal rotation [16–18] will be present at baseline measures in those that develop RCS.

## Methods

### Study design overview and participants

This is a prospective cohort study design. Individuals in high-risk occupations *without* current shoulder pain or injury will be recruited and followed for 2 years. All participants will be assessed at baseline with the full physical protocol and will be contacted for follow up questionnaire assessments every 3 months for the study period. Recruitment is expected to begin in April 2025 and continue until the summer of 2029. Data collection will be completed in the summer of 2031 (two years following final recruitment), and results will be expected by the end of 2031. All procedures have been approved by the University of Saskatchewan’s research ethics board.

As the proposed design and analyses are novel, in the absence of similar prospective research using continuous kinematic variables as independent variables, a simple, robust approach was chosen for the sample size calculation. Using an *a priori* independent t-test sample calculation, we have identified a minimum sample size of 238 individuals in high-risk occupations to detect a significant mean difference of 7° (standard deviation 9°) in scapular angles between individuals who develop RCS and those who do not at a 5% level of significance and with 80% power. We assume an incidence of RCS of 6% in this high-risk population [19]. We applied a variance inflation factor to account for the inclusion of potential confounders in our models as per Hsieh et al [20]. Assuming a low confounding effect, and accounting for a conservative 85% retention, a total sample size of 292 individuals will be approached for recruitment.

Adults (equal to or over 18 years) will be recruited through several high-risk occupations. Occupations will be chosen to ensure equitable recruitment across sex. Gender will be collected with the expanded gender questionnaire [21] and sub analyses will be performed if sufficiently powered. Construction workers [22], hairdressers [23], nurses and nurse aids [24], farmers [25], maintenance workers and caretakers [26] will be targeted, based on high RCS prevalence. Exclusion criteria will include current shoulder pain; history of shoulder injury that resulted in a) a clinician visit (primary care or physical therapy), or b) stoppage in regular activities (work or sport/leisure) for 1 month or more; current upper limb arthritis; history of stroke; previous shoulder or chest surgery; carpal tunnel syndrome; and cervical dysfunction radiating to the shoulder.

### Baseline session

All participants will be assessed at baseline at the Shoulder Health and Ergonomics (SHER) Research Lab in Saskatoon, Saskatchewan. Upon arrival, each participant will provide written informed consent, witnessed by the researchers. Each person will then be screened with pain provocation tests (see *Follow up procedures* section), and a positive result (i.e., pain) on any test will warrant exclusion. Participant characteristics will also be recorded, such as age, height, weight, biological sex at birth, and gender. Participants will then complete several validated self-reported questionnaires (Quick Disabilities of the Arm, Shoulder and Hand (QuickDASH) [27]; Shoulder Pain and Disability Index (SPADI) [28,29]; International Physical Activity Questionnaire [30]; Nordic Musculoskeletal Discomfort questionnaire [31], and Fear Avoidance Behaviour Questionnaire [32] to describe current function and activity levels. Additionally, they will complete questionnaires about individual characteristics (occupation, workload (FTE), dominance, education, and co-morbidities such as diabetes [33], and about their typical work exposures [31], which are detailed below (outcome measures summary). Using self-reports of work exposures is a standard approach in prospective injury research and is expected to sufficiently describe relevant regular exposures [6,31]. Four bilateral isometric strength tests will also be performed to assess baseline strength of key scapular muscles, as per Michener et al. (2005) [34], using a handheld dynamometer [35].

**Fig 1:**
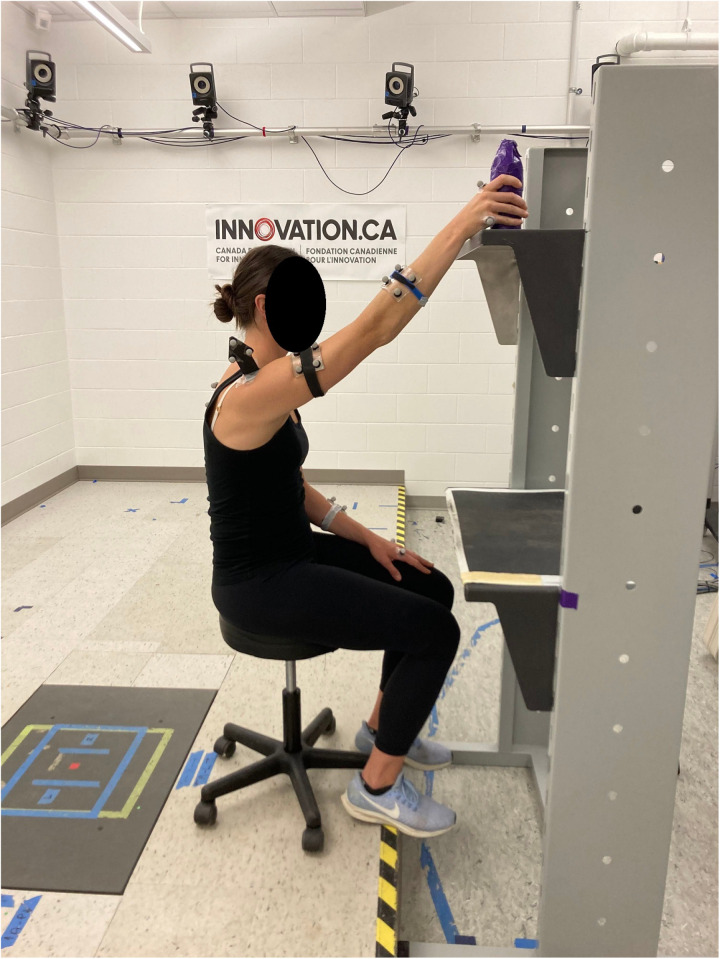
The overhead reach with the right arm.

At the baseline lab testing session, scapular and humeral kinematics will be assessed during a standardized unilateral overhead reaching motion (Fig 1). This task was chosen as it was previously used in research in the SHER lab with individuals with RCS laying the foundation for this research [16]. Notable alterations were found in this task in that study, as well as in previous research with breast cancer survivors [17,36,37]. The overhead reach is part of a standardized protocol that the research group has validated [38]. Each participant will sit in front of a set of shelves. Holding a 1 kg bottle in their hand, they will reach up to a high shelf set to 1.5 meters off the ground. The overhead reach will be performed 3 times with each arm.

Three-dimensional motion of the thorax, scapulae, and humeri will be tracked using a Vicon optoelectronic infrared camera system (Vicon, Oxford, UK). Individual markers and marker clusters will be placed on the torso and upper extremity as per our published methods [17,39,40] and following the International Society of Biomechanics recommendations [41].The scapulae will be tracked with the acromial marker cluster using a double calibration method, which has been validated in previous work [42]. Marker positions will be sampled at 100 Hz.

#### Kinematic data reduction.

Marker trajectory data will be processed with custom MATLAB® scripts. Thoracohumeral elevation and scapular kinematics (internal/external rotation, upward rotation, tilt) will be calculated from marker trajectory data based on the International Society of Biomechanics standards [41]. Scapular orientations at specific humeral elevation levels will be extracted [38,43,44].

#### Follow up procedures.

Participants will be contacted via email or phone every 3 months for 2 years to assess any development of shoulder pain or injuries and directed to re-input select individual information (i.e., occupation title, presence of co-morbidities). If their occupation changes, they will be asked to re-assess their self-reported work exposures by repeating the exposures survey online. If they indicate a development of symptoms at any follow up, participants will be contacted via phone for symptom screening. If participants are determined to be experiencing pain or injury, they will be invited back to the SHER Lab for a short clinical evaluation. At the in-person clinical assessments, participant characteristics and occupation details will again be recorded. Participants will also repeat the QuickDASH and SPADI questionnaires, will be asked about current and previous shoulder pain, pain when raising their arm, and screened with four provocative tests (painful arc, Hawkins-Kennedy, infraspinatus, empty can) [36]. If they experience regular pain (4 or more days in the past week), pain when raising their arm, and positives (i.e., pain) on 1 or more tests [31,45,46] they will be placed in the RCS group for analysis.

### Statistical analysis

All statistical analyses will be performed using SAS/STATA. To assess if scapular kinematics are risk factors for RCS, a two-step analysis will be performed. First, scapular kinematics will be compared between participants who develop symptomatic RCS (see Table 1 for criteria) and those who do not with an independent samples t-test (p < .05). Second, while considering a dichotomous outcome (presence or absence of RCS), we will fit univariable and multivariable logistic and Cox regression models. The strength of association will be assessed by the odds ratio and hazard ratio, respectively, along with 95% confidence intervals. For survival analyses, the time-to-event of interest (i.e., incident RCS) will be computed based on the difference between baseline data collection point (time of origin) and follow-up time to the event occurrence. The follow-up time for censored cases will be computed based on the difference between baseline and no event occurrence by study end or last follow-up point. We will employ Cox regression analyses for the incidence of RCS over time. Multivariable models will be fitted to include potential confounders, which will be selected based on purposeful selection as per Hosmer [48] This includes selection based on statistical significance, clinical importance, and the impact of removing that variable has on the variables remaining in the model.

**Table 1:** Description of dependent and predictor variables to be included in the analysis.

Variable	Description
**Dependent variable**	
**Primary:** the presence of clinically screened symptomatic RCS at any time in the follow up period	1) Reports of regular pain at follow up (4 or more days in the past week); 2) Pain with raising the arm; and 3) Pain on 1 or more clinical provocation tests
**Predictor variables**	
**Primary**: Scapular kinematics at select humeral elevation levels (30° increments)	Internal/external rotation Upward/downward rotation Anterior/Posterior tilt
Age	
Biological Sex	Female/Male
Gender	Man; woman; Trans man; Trans woman; Genderqueer; Other (specified by participant)
Body mass index (BMI)	Ratio of body mass and height (kg/m^2^)
Occupation	
Workload	% FTE
Experience in current occupation	In years
Hand dominance	
Shoulder muscle strength	Upper trapezius Middle trapezius Lower trapezius Serratus anterior
Highest level of education	Less than high school; completed high school; Completed journeyperson certificate/diploma; Completed university degree
Co-morbidities [33,47]	Diabetes, hypertension, depression, anxiety
Typical work exposures at baseline and follow up [31]	1) Work organization (pace, independence, temperature) 2) Biomechanical (ratings of perceived exertion, arms above shoulders, sustained abduction, high repetition, use of hand tools, exposure to vibration, hand behind back) 3) Psychosocial (coworkers support, job satisfaction)

## Conclusion

This research represents a crucial and important step for understanding shoulder musculoskeletal health. This project is an opportunity to yield critical, unprecedented insights into the development of symptomatic RCS. At present, the links between potentially harmful biomechanics and RCS are based solely on cross-sectional data of individuals already diagnosed with RCS. Prospective assessment of the role of biomechanics as a risk factor for RCS will pave the way for innovative, evidence-based treatment and prevention strategies. This research will also inform necessary future work on appropriate prevention strategies and novel rehabilitation approaches to prevent injuries and improve quality of life across the population.
